# Causes of Mortality for Indonesian Hajj Pilgrims: Comparison between Routine Death Certificate and Verbal Autopsy Findings

**DOI:** 10.1371/journal.pone.0073243

**Published:** 2013-08-21

**Authors:** Masdalina Pane, Sholah Imari, Qomariah Alwi, I Nyoman Kandun, Alex R. Cook, Gina Samaan

**Affiliations:** 1 Hajj Health Subdirectorate, Ministry of Health, Jakarta, Indonesia; 2 National Health Institute Research and Development, Ministry of Health, Jakarta, Indonesia; 3 Field Epidemiology Training Program, Ministry of Health, Jakarta, Indonesia; 4 Saw Swee Hock School of Public Health, National University of Singapore and National University Health System, Singapore, Singapore,; 5 National Centre for Epidemiology and Population Health, Australian National University, Canberra, Australia; 6 Department of Statistics and Applied Probability, National University of Singapore, Singapore, Singapore; 7 Duke-NUS Graduate Medical School Singapore, Singapore, Singapore; 8 Communicable Disease Centre, Tan Tock Seng Hospital, Singapore, Singapore,; Alberta Provincial Laboratory for Public Health/ University of Alberta, Canada

## Abstract

**Background:**

Indonesia provides the largest single source of pilgrims for the Hajj (10%). In the last two decades, mortality rates for Indonesian pilgrims ranged between 200–380 deaths per 100,000 pilgrims over the 10-week Hajj period. Reasons for high mortality are not well understood. In 2008, verbal autopsy was introduced to complement routine death certificates to explore cause of death diagnoses. This study presents the patterns and causes of death for Indonesian pilgrims, and compares routine death certificates to verbal autopsy findings.

**Methods:**

Public health surveillance was conducted by Indonesian public health authorities accompanying pilgrims to Saudi Arabia, with daily reporting of hospitalizations and deaths. Surveillance data from 2008 were analyzed for timing, geographic location and site of death. Percentages for each cause of death category from death certificates were compared to that from verbal autopsy.

**Results:**

In 2008, 206,831 Indonesian undertook the Hajj. There were 446 deaths, equivalent to 1,968 deaths per 100,000 pilgrim years. Most pilgrims died in Mecca (68%) and Medinah (24%). There was no statistically discernible difference in the total mortality risk for the two pilgrimage routes (Mecca or Medinah first), but the number of deaths peaked earlier for those traveling to Mecca first (p=0.002). Most deaths were due to cardiovascular (66%) and respiratory (28%) diseases. A greater proportion of deaths were attributed to cardiovascular disease by death certificate compared to the verbal autopsy method (p<0.001). Significantly more deaths had ill-defined cause based on verbal autopsy method (p<0.001).

**Conclusions:**

Despite pre-departure health screening and other medical services, Indonesian pilgrim mortality rates were very high. Correct classification of cause of death is critical for the development of risk mitigation strategies. Since verbal autopsy classified causes of death differently to death certificates, further studies are needed to assess the method’s utility in this setting.

## Introduction

Each year, Muslims from all over the world undertake the Hajj pilgrimage to and in Saudi Arabia [1]. In recent years, over 2 million people from 140 countries undertook the Hajj annually, including over 200,000 people from Indonesia, the world’s most populous Muslim majority country. Performance of the Hajj and its rites is physically demanding [2]. Extreme physical stressors such as sun exposure, heat (37^°^C during the day and 20^°^C at night), thirst, crowding, steep inclines and traffic congestions over a prolonged period of time (40 days per pilgrim over the 70-day Hajj season) increase health risks. Since pilgrims tend to be older and many have medical comorbidities [3], these factors exacerbate existing risk for ischemic and congestive cardiovascular disease, fluid and electrolyte abnormalities, and respiratory and other infectious diseases including emerging diseases such as Middle East Respiratory Syndrome Coronavirus [4].

In the last two decades, the mortality rate of Indonesian pilgrims, excluding years in which disasters such as stampedes occurred, fluctuated between 200–380 deaths per 100,000 persons during the ten-week Hajj period [5]. Few countries have published pilgrim mortality rates, but compared to where they are available, the Indonesian rate is much higher [6]. For example, in 1998, the Hajj mortality rate amongst Isfahani pilgrims from Iran was 13 per 100,000 pilgrimages [7]. In 2004, the mortality rate for all Iranian pilgrims was 47 per 100,000 pilgrimages, and in 2005, 24 per 100,000 [6]. Even compared to the yearly mortality rate in Indonesia, the mortality rate for Hajj pilgrims ranged between 1,765 and 3,353 per 100,000 per year; by comparison, the Indonesian estimated national crude death rate was 700 per 100,000 in 2003 [8].

Since 1950, Indonesian authorities have provided medical services to Hajj pilgrims. Pre-departure health screening and vaccination as well as temporary medical clinics staffed by Indonesian doctors in Saudi Arabia were introduced progressively. Mortality surveillance was also established to reduce mortality and aid in administrative processes for life insurance claims. Prior to 2008, the patterns, causes of death and factors associated with mortality were not well understood for Indonesian pilgrims. Data collected were limited to basic demographic characteristics and general cause of death. Cause of death could only be obtained from the hospital death certificate or the flight doctor’s records if a death occurred in the community. These were based on clinical examination and any available laboratory tests as documented in the patient medical record, but they lacked detailed information about the cause of death or the patient’s pre-existing conditions.

Given the hugely elevated mortality risk relative to the general Indonesian population, information on cause of death and underlying health conditions is critical to enhance pilgrim health management and to prevent excess mortality. In 2008, the Ministry of Health introduced an additional surveillance tool – verbal autopsy – to better understand the causes of pilgrim mortality. Verbal autopsy allows for the systematic investigation of probable causes of death through structured questionnaires [9]. The World Health Organization (WHO) advocates the use of verbal autopsy in situations where only a fraction of deaths occur in hospitals or in absence of vital registration systems [10]. Previous studies have shown that verbal autopsy improves diagnosis of cause of mortality and the method continues to be used in various countries [11–13].

This study describes the findings from the mortality surveillance conducted during the Hajj in 2008; the year in which verbal autopsy was introduced. We explore both person-specific and site-specific factors associated with mortality, and compare the cause of death from the routine death certificate to the newly introduced verbal autopsy method.

## Methods

Ethics approval for the study was obtained from the Ministry of Health National Institute of Health Research and Development ethics review committee with approval number LB.03.04/KE/4687/2008. Written consent was not obtained from the patients involved, but this was waived by the ethics review committee as mortality data were analysed anonymously.

### Study population

In 2008, 206,831 Indonesians undertook pilgrimage during Hajj. The majority were from Java (58%) and Sumatra (24%) islands, with small proportions from Kalimantan (6%), Sulawesi (4%) and other (8%) islands. The majority of pilgrims, anecdotally reported to be 95%, joined one of the government-sponsored Hajj pilgrimage travel services that include 40 days of travel. The remainder joined private more expensive pilgrimage travel services that are of shorter duration (25 days) and provide better accommodation and services.

### Health services and surveillance

A number of preventive and curative healthcare services were available to all Hajj pilgrims prior to and during their travels in Saudi Arabia. Pre-departure, each Indonesian pilgrim was required to visit a government healthcare facility for a medical check-up and to receive a pocketbook outlining their health conditions, medications and vaccination status. Pilgrims were mandated to receive meningococcal vaccine and were advised to receive influenza vaccine before their departure. Flights were chartered by the Indonesian government to accommodate 300–450 pilgrims. Each flight had one doctor and two nurses to accompany the pilgrims. These healthcare workers treated, triaged and conducted health surveillance for pilgrims. Hospitalizations and incidental reporting of any deaths occurring outside a healthcare facility were notified daily to the Indonesian public health team based in Saudi Arabia during the Hajj.

Saudi authorities made first aid posts, health centres and hospitals available. In Mecca, six hospitals were set up during pilgrimage, while in Medinah, pilgrims could access established hospitals. In addition to the Saudi facilities, Indonesian health authorities set up field hospitals in Mecca and Medinah specifically for Indonesian pilgrims. Indonesia also sent 306 specialist doctors including internists, pulmonologists, cardiologists and psychiatrists, public health workers, nurses, pharmacists and sanitarians to support the pilgrimage. All deaths were reported from the flight doctor or the Saudi hospital to the Indonesian public health team located in Saudi Arabia during the Hajj.

### Data collection and analysis

Mortality data collected by the Indonesian public health team were entered into a database for data analysis. Standard variables in the database included name, age, sex, home address, employment, flight group, time and place of death, date of arrival into Saudi Arabia, cause of death as per the hospital or flight doctor death certificate.

In 2008, the Indonesian Ministry of Health mandated a verbal autopsy form to be filled out by the flight doctors accompanying pilgrims. The verbal autopsy form was developed based on the standards established by WHO and adapted to Hajj pilgrimage needs [14]. The verbal autopsy form obtained detailed information about medical history, signs and symptoms, and other circumstances regarding the death from family or friends who travelled with the deceased. In most cases, interviews were conducted with a combination of the treating physician, the deceased person’s spouse or pilgrims in the same flight group. The form aimed to increase the specificity of the cause of death by elucidating medical history and recent events.

The flight doctors were trained in administering the verbal autopsy form prior to departure from Indonesia and were required to complete it within the week of a pilgrim’s death. Once the form was completed, the Indonesian public health team stationed in Saudi Arabia sent it to the Ministry of Health in Indonesia for analysis and determination of cause of death. The form was analysed separately by two trained staff at the Ministry of Health to determine the cause of death. If discordant, the staff compared their analyses to achieve consensus. The cause of death based on this verbal autopsy method was then recorded in the database and compared to that reported by the hospital or flight doctor death certificate.

We used counts and proportions to describe demographic characteristics of Indonesian pilgrims and fatalities. The Chi-Square test and Chi-Square test for trend were used for the analysis of categorical data. To compare the two methods for establishing cause of death (verbal autopsy and death certificate), we compared the percentages for each cause of death category using McNemar’s test.

## Results

### Indonesian pilgrims for the Hajj in 2008

All 206,831 Indonesian pilgrims were aged 18 years or more, where the majority (59%) were aged between 41 and 59 years (Table 1). Most pilgrims were female (55%, Table 1). According to the pre-embarkation medical assessment, 28% of pilgrims were classified as high risk due to underlying health conditions such as diabetes, hypertension, other chronic diseases or if they were 60 years or older.

**Table 1:** Demographic characteristics of Indonesian population in 2008 and Indonesian pilgrims during the Hajj in 2008*.

Characteristic	N (%)	Indonesian Population (%)^#^
Gender		
Male	93,600 (45)	115,000,000 (50)
Female	113,000 (55)	113,000,000 (50)
Age groups		
18-40 years	40,400 (20)	79,900,000 (56)
41-49 years	60,500 (29)	28,600,000 (20)
50-59 years	60,400 (29)	18,300,000 (13)
≥60 years	45,600 (22)	16,500,000 (12)
Employment		
Private business	68,500 (33)	^
Housewife	58,900 (29)	32,800,000 (37)
Civil servant	37,600 (18)	^
Farmer	26,700 (13)	5,990,000 (7)
Student	2,070 (1.0)	13,200,000 (15)
Police/military	1,660 (0.8)	^
Unknown	11,400 (5)	8,700,000 (10)
High health risk pilgrims	58,500 (28)	Not applicable

* Figures rounded to three significant numbers.

# Indonesian population gender and age-group data based on projections from 2005 national census. Employment data obtained from Bureau of Statistics survey conducted in 2008. Muslims comprise 85% of the total Indonesian population.

^ Disaggregated data not available. Total for the three groups = 28,200,000.

**Figure 1:**
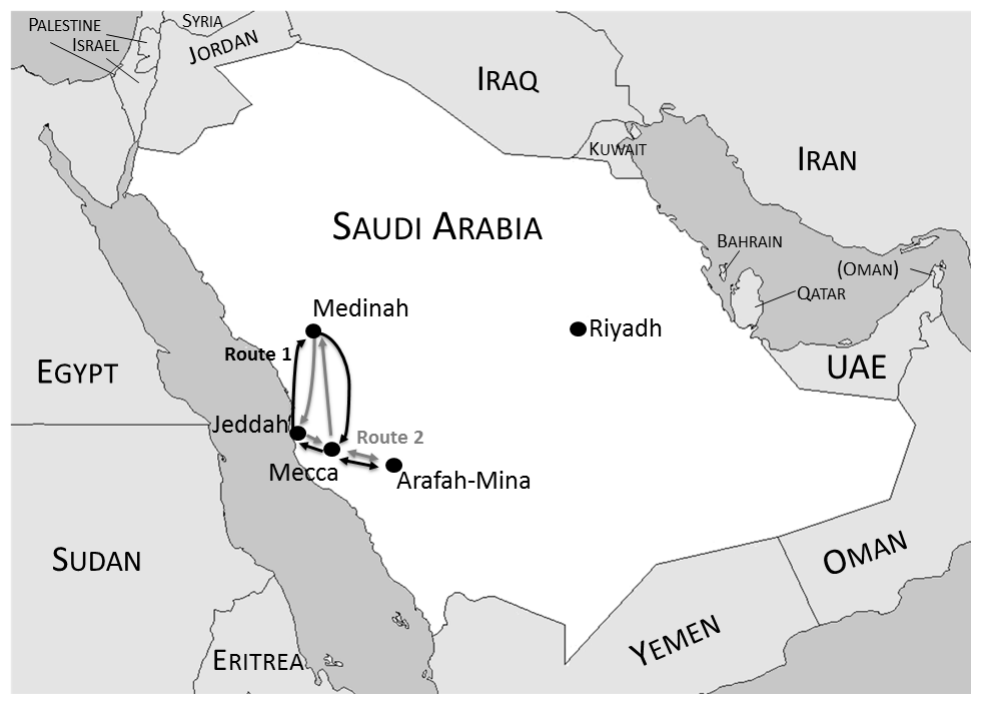
Routes of travel for Indonesian pilgrims, arrival in and departure from Jeddah.

Pilgrims arrived into Saudi Arabia in phases. The first group arrived in Saudi Arabia on 5 November 2008, the last on 2 December 2008. The groups also return to Indonesia in phases, with the first group arriving back on 14 December 2008, and the last one on 10 January 2009. Pilgrims traveled around Saudi Arabia in one of two routes: Jeddah → Medinah → Mecca → Arafah-Mina → Mecca → Jeddah, or Jeddah → Mecca → Arafah-Mina → Mecca → Medinah → Jeddah, with some returning to Indonesia directly from Medinah (Figure 1). Even though the Hajj period is ten weeks, duration of travel for pilgrims on either route was 40 days, of which 22 days were spent in Mecca, 5–6 days in Arafah-Mina, 8–9 days in Medinah and 1–2 days in transit in Jeddah.

### Mortality

In 2008, 446 Indonesian pilgrims died during the Hajj in Saudi Arabia. The overall mortality rate was 216 deaths per 100,000 pilgrimages. Mortality rates were highest in those ≥60 years of age (722 per 100,000 pilgrims), and rates significantly increased with increasing age (p<0.01, Table 2). Mortality rates were higher in males (296 deaths per 100,000 pilgrims) compared to females (150 deaths per 100,000 pilgrims, p<0.01, Table 2). For the 4 deaths in 18-40 year old age-group, 2 were male and 2 were female. According to the death certificates, three died due to cardiac arrest and one due to asphyxia in a patient with active tuberculosis.

**Table 2:** Mortality rates of Indonesian pilgrims in 2008, total deaths = 446.

	Deaths	Rate per 100,000 pilgrimages (95%CI)	p-value
Age Group			
18-40	4	10 (0.2, 20)	
41-49	25	41 (25, 58)	
50-59	88	146 (115, 176)	
≥60	329	722 (644, 800)	p<0.01
Sex			
Male	276	296 (261, 331)	p<0.01
Female	170	150 (127, 172)	

**Figure 2:**
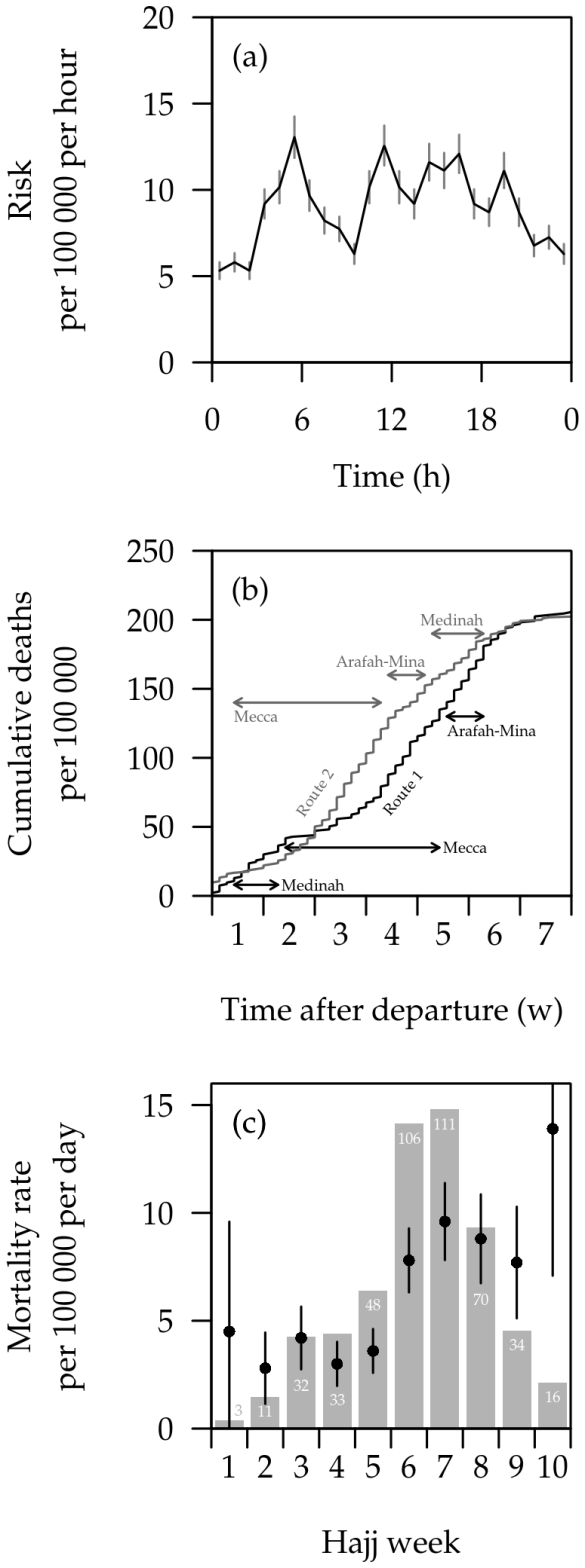
Timing of deaths on the Hajj. (a) Hour of death, as risk per 100,000 pilgrim-hours, with 95% confidence intervals. (b) Cumulative number of deaths over course of the Hajj, for two routes, per 100,000 pilgrimages. 93,357 pilgrims took route 1, and 113,474 route 2. Both routes start and end in Jeddah. (c) Mortality rate over the duration of the Hajj. Numbers of deaths are presented in grey bars; rate per 100,000 pilgrim-days and 95% confidence intervals are presented in black.

Most deaths occurred in Mecca (n=305, 68%), followed by Medinah (n=106, 24%) and Jeddah (n=35, 8%). The majority (57%) of deaths occurred in hospital, but a large proportion of deaths also occurred in pilgrims’ apartments/sleeping areas (36%). Most (77%) deaths occurred during pilgrims’ active hours between 5am and 9pm: mortality rates were higher in the afternoon (possibly due to the heat) and early morning (Figure 2a).

Weekly mortality rates increased in week 6, exceeding the expected crude mortality rate (CMR) of 5 per 100,000 per day [15], and remained high until the end of the 10-week Hajj period (Figure 2b). In week 8, the number of deaths started to decrease. However, since the overall number of pilgrims also decreased, the mortality rates remained very high since some of the Indonesian pilgrims who were hospitalized died in the later weeks of the Hajj.

For pilgrims on Route 1, the number of deaths increased sharply four weeks after arrival into Saudi Arabia by which time the pilgrims had already reached Mecca (Figure 2b). For pilgrims on Route 2, the number of deaths peaked in the third week after their arrival into Saudi Arabia, at which stage the pilgrims were in Mecca (Figure 2c). The number of deaths remained large thereafter until the end of the Hajj for those undertaking Route 2. There was no statistical difference in the number of deaths occurring for pilgrims undertaking Route 1 (200 out of 93,357 pilgrims) compared to those on Route 2 (231 out of 113,474, p=0.72). However, the trends from week to week during the Hajj period were significantly different between pilgrims on Route 1 compared to those on Route 2 (χ^2^ for trend=9.23, p=0.002).

### Cause of death

Most deaths were due to cardiovascular diseases and respiratory diseases (Table 3). A greater proportion of deaths were attributed to cardiovascular disease by the flight doctor or hospital death certificate (66%) compared to the cause of death ascertained using the verbal autopsy method (49%, p<0.001). Significantly more deaths had unspecified cause based on the verbal autopsy method (10%, versus 0, p<0.001). As part of the demographic data collected for each pilgrim, height and body mass were recorded. Based on the hospital/flight doctor death certificate, 38 of the 446 (9%) pilgrims who died had body mass index ≥27.5 indicating obesity for Asian body types [16]. Of these, 26 died due to cardiovascular disturbances, 7 due to respiratory illness, 2 due to metabolic disturbances, 2 had undefined sudden death and 1 due to trauma (neck fracture).

**Table 3:** Comparison of cause of death according to hospital/flight doctor death certificate and verbal autopsy method, N=446.

	Death certificate issued by hospital/flight doctor, N (%)	Cause of death based on verbal autopsy, N (%)	p-value
Cardiovascular diseases	292 (66)	218 (49)	<0.001
Respiratory diseases	126 (28)	156 (35)	0.007
Neurological diseases	6 (1)	2 (0.5)	0.125
Cancer (neoplasm)	6 (1)	0	0.030
Injuries/Trauma	4 (0.9)	0	0.125
Gastrointestinal diseases	2 (0.4)	0	0.50
Communicable diseases	2 (0.4)	8 (2)	0.030
Metabolic diseases	2 (0.4)	2 (0.5)	1
Mental disturbance	0	5 (1)	0.063
Other	6 (1)	12 (3)	0.15
Ill-defined/unspecified	0	43 (10)	<0.001
Total	446	446	

## Discussion

This study describes the demographics, patterns and causes of mortality in Indonesian pilgrims in 2008. Nearly one third of pilgrims undertaking the Hajj in 2008 were considered high risk due to underlying health conditions or their age. Mortality rates were found to be greatest in males and in those aged ≥60 years, in whom most deaths were attributed to cardiovascular and respiratory diseases. Studies from other countries with pilgrims found similar trends, including a preponderance in male mortality [4,17]. These findings highlight the special characteristics of the Hajj compared to other mass gathering events. Many Muslims wait decades for the opportunity to perform the Hajj, and by the time they receive the chance, they may have a multitude of age-related health concerns [18].

Correct classification of deaths is critical to target preventive interventions and provide health services [14]. This study compared cause of death according to the flight doctor and death certificate records to the newly introduced verbal autopsy method. Fewer deaths were attributed to cardiovascular diseases using verbal autopsy but this method resulted in a greater number of deaths having ill-defined cause of death. Verbal autopsy method may have reduced misclassification by removing pressure from clinicians having to extrapolate cause of death in situations where it may have been ill-defined or unclear. However, this hypothesis warrants further investigation. Since the verbal autopsy method is dependent on the skills of the field personnel collecting the data, the timing to limit recall bias and the method is most suited to diseases with specific symptoms and presentation [10,19], the use of verbal autopsy for Hajj mortality surveillance should be further evaluated.

Based on both the death certificates and verbal autopsy categories, cardiovascular disease was the leading cause of Indonesian pilgrim mortality in 2008. Performance of obligatory rites during the Hajj constitutes stressful exercise which is not generally recommended by doctors for those with ischemic heart disease, hypertension or heart failure as such exercise may increase the risk of heart attacks [20]. This risk may be further elevated in the heat, where dehydration may lead to increase in body temperature and heart rate, and a decrease in cardiac output [21]. An Iranian study showed that when patients with severe cardiovascular disease were prohibited from attending the Hajj and other patients with cardiovascular disease were provided with appropriate medications and monitoring during pilgrimage, mortality rates were significantly lower than those for other pilgrims [7]. This supports the need for careful pre-departure health screening, exclusion of the severely ill, provision of appropriate drug therapies or increased physical exercise prior to departure, and monitoring during pilgrimage to reduce mortality.

One-third of Indonesian pilgrim mortality was attributed to respiratory diseases. Pneumonia is a common illness that is life-threatening to the elderly, especially those with comorbidities such as diabetes or hypertension [4]. A number of studies have shown that pneumonia is the primary cause of critical illness during the Hajj and that etiologies include Gram-negative organisms, *Streptococcus pneumoniae*, and *Mycobacterium tuberculosis* [2,18,22]. For tuberculosis patients, the physical stressors of the Hajj may increase the risk of severe illness and mortality, and the intense crowding during the pilgrimage may increase the risk of disease transmission. A recent study found that 10% of Malaysian pilgrims had a significant increase in immune response to QuantiFERON tuberculosis assay antigen post-Hajj compared to pre-Hajj [23]. Since Indonesia is a high-burden country for tuberculosis [24], pre-departure screening should continue to exclude those with active disease. Other potential public health measures to reduce mortality due to respiratory diseases include increasing coverage of influenza vaccine and pneumococcal vaccine. Such measures were applied by Iranian public health authorities in 2005, which halved the incidence of respiratory diseases and decreased the mortality rates from 47 per 100,000 in 2004 to 24 per 100,000 in 2005 [6].

Deaths amongst Indonesian pilgrims traveling on Route 2 who went to Mecca first peaked earlier than those traveling on Route 1. Most deaths among Indonesian pilgrims occurred in the middle-latter weeks of the Hajj period during the stay in Mecca and afterwards in Arafah-Mina. Obligatory rites conducted at holy sites in Mecca and Arafah-Mina are known to involve intense physical activity [7]. These may have been too strenuous for some pilgrims, especially older or relatively sedentary pilgrims. Surprisingly, 36% of deaths occurred in the accommodation provided to pilgrims during the Hajj. This highlights that despite the presence of an accompanying health team, not all patients were referred to hospital prior to critical stages of illness. One limitation of this study is that further details about deaths occurring in accommodation were not available for analysis. Lack of data limited other important analyses including deaths by health risk status and type of travel service (government or private) used. The rates of mortality as well as causes of death may differ based on these categories and may impact recommendations for intervention.

## Conclusions

Indonesian pilgrims suffer high mortality rates despite pre-departure screenings, accompanying medical teams and the availability of specialized health services during the Hajj. This study highlights the importance of surveillance during the Hajj to understand the health risks and strengthen the evidence-base on which policy can be developed [2]. Further studies are needed to assess verbal autopsy’s utility in this setting. The role of the accompanying health teams as first responders needs to be reviewed to determine how they can best reduce Indonesian pilgrim mortality. An evaluation of the current mortality surveillance system is also warranted to ensure that the data collected appropriately serves the public health purpose of reducing pilgrim mortality. Lastly, lessons need to be learnt from other countries including their Hajj mortality patterns and risk mitigation strategies.
